# A distal ileum malignant peripheral nerve sheath tumor causing intussusception in a patient in China: a case report

**DOI:** 10.1186/s12957-017-1101-z

**Published:** 2017-01-19

**Authors:** Lin-Bo Zhu, Peng-Fei Li, Wei-Hua Xiao, Peng-Bin Zhang, Jun-Qiang Li, Ming-Fei Sun

**Affiliations:** 1 Department of General Surgery, Beilun District People’s Hospital, The First Affiliated Hospital, College of Medicine, Beilun Branch of Zhejiang University, No.1288, Lushan East Road, Ningbo, Zhejiang 315800 China; 2 Department of Pathology, Beilun District People’s Hospital, The First Affiliated Hospital, College of Medicine, Beilun Branch of Zhejiang University, Ningbo, Zhejiang 315800 China

**Keywords:** Malignant peripheral nerve sheath tumor, Small bowel

## Abstract

**Background:**

Malignant peripheral nerve sheath tumors (MPNSTs) arise from a peripheral nerve or display nerve sheath differentiation. Most MPNSTs typically originate on the trunk, extremities, head, neck, and paravertebral regions. Gastrointestinal MPNSTs are rare entities with only 10 cases reported worldwide in the literatures.

**Case presentation:**

Here, we report the first Chinese case of a malignant peripheral nerve sheath tumor of the distal ileum presenting as intussusception. A 53-year-old female patient without pathological antecedent for neurofibromatosis was admitted with pain in the right lower abdomen and multiple episodes of vomiting for 1 week. Preoperative diagnosis was intussusception with a contrast-enhanced computed tomography scan (CECT) of the abdomen showing characteristic target sign. Due to difficulty reducing the ileum-colon intussusception, right hemicolectomy with ileocolostomy was performed. Histopathology was suggestive of low-grade MPNST. The patient received postoperative care and was followed up for 9 months. There is no sign of tumor recurrence and metastatic disease.

**Conclusions:**

This case is unique in terms of a rare tumor presenting with unusual complication.

## Background

Malignant peripheral nerve sheath tumors (MPNSTs) are defined as any tumor arising from a peripheral nerve or exhibiting nerve sheath differentiation. Most MPNSTs are located along major nerve trunks, commonly arising on the body trunk, extremities, head, neck, and paravertebral regions. MPNSTs arising from nerves of the small intestinal wall are extremely rare, with only 10 cases reported worldwide. In this report, to the best of our knowledge, we describe the first Chinese case of a malignant peripheral nerve sheath tumor of distal ileum presenting as intussusception in a patient.

## Case presentation

**Fig. 1:**
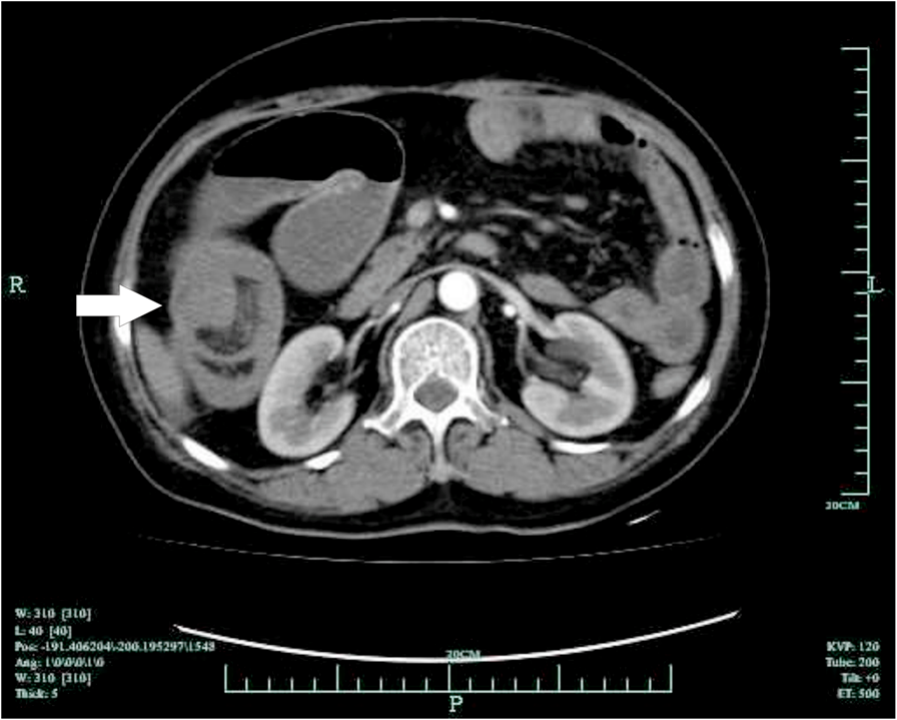
Contrast-enhanced computed tomography scan of the abdomen showing intussusception in the ascending colon with a smooth enhancing 3.5 cm mass suspicious for neoplasm (*white arrow*)

**Fig. 2:**
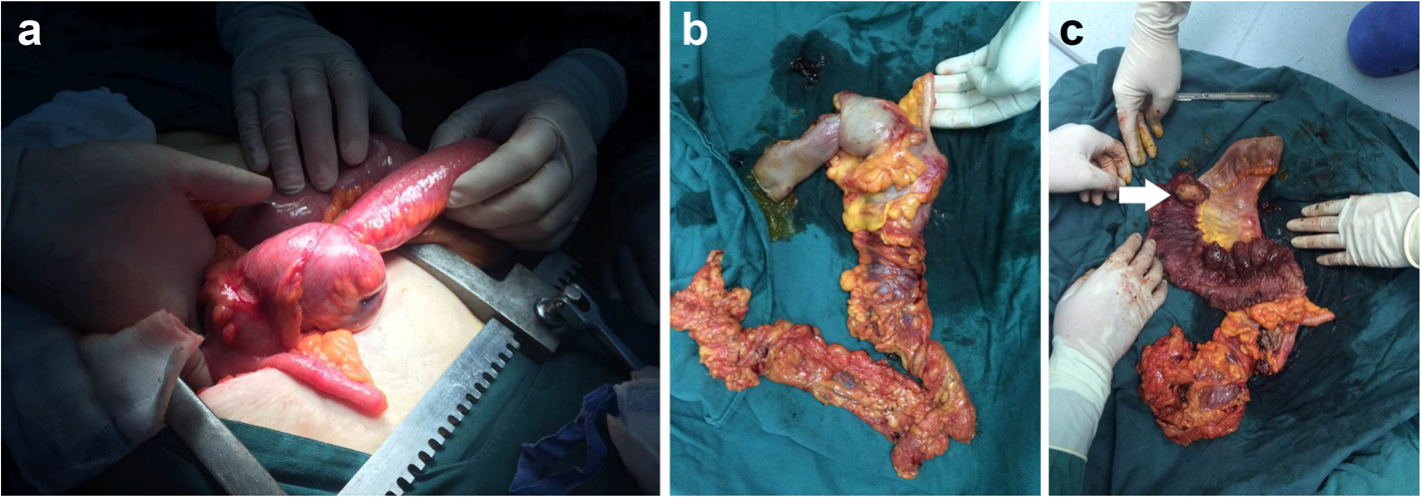
**a** Difficulty reduction of ileum-colon intussusceptions on emergent exploratory laparotomy. **b** A right hemicolectomy specimen. **c** A 4.0 × 3.5 × 2.3 cm submucosal tumor of distal ileum (*white arrow*)

**Fig. 3:**
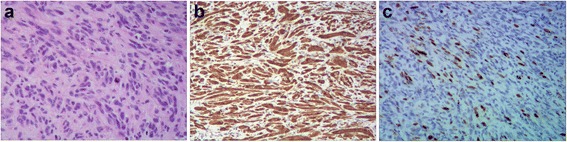
**a** The tumor showing bundles and fascicles of atypical spindle cells with hyperchromatic nuclei and indistinct cytoplasm (HE, ×200). **b** S-100 staining showing diffuse positive reaction. **c** IHC staining for Ki67-labeling index 15~20%

A 53-year-old female patient presented to the First Affiliated Hospital of Beilun Branch of Zhejiang University China on September 2015 complaining of pain on the right side of her abdomen associated with abdominal distension and multiple episodes of vomiting for 1-week duration. She denied changes in bowel habits, blood in stools, and past abdominal operations. Her family history was insignificant for neurofibromatosis type 1. On abdominal examination, she exhibited abdominal guarding, but no palpable mass was found. Abdominal CECT demonstrated intussusception in the ascending colon with a smooth enhancing 3.5 cm mass suspicious for neoplasm (Fig. 1). Colonoscopy was deferred due to significant exacerbation of pain and worsening peritonitis. The patient subsequently underwent emergent exploratory laparotomy. Due to difficulty reducing the ileocolic (Fig. 2a), right hemicolectomy with ileocolic anastamosis was performed (Fig. 2b). Exploration of the abdomen revealed no carcinomatosis. The intraoperative frozen section displayed spindle cell tumor of the small bowel. Although there seemed to be no apparent lymphadenopathy in the mesentery, we resected the mesentery to include the potential lymph node metastases. The resected specimen revealed a 4.0 × 3.5 × 2.3 cm submucosal mass of the distal ileum (Fig. 2c). Macroscopic examination demonstrated a gray-white appearance of the tumor cross section, without hemorrhage and necrosis. Histologically, the tumor was composed of bundles and palisading arrangement of malignant spindle cells that extended into the muscularis mucosa (Fig. 3a). The tumor had dense cellularity with significant mitotic activity of approximately 10 mitoses per 10 high-power fields. The surgical margins were negative for the tumor. On immunohistochemical staining, the tumor was positive for S-100 (Fig. 3b) and CD34, but negative for CD117, DOG-1, SMA, AE1/AE3, HMB45, and PNL-2. The Ki67 labeling index was approximately 15–20% (Fig. 3c). On the basis of these pathological findings, the tumor was identified as a low-grade malignant peripheral nerve sheath tumor (MPNST).

The patient declined to undergo further genetic analysis and chemotherapy. She had a good postoperative course and was discharged 2 weeks after surgery. She presented with diarrhea, fever, and moderate anemia at 3 and 7 months post-operation. Abdominal CECT demonstrated no sign of tumor recurrence and metastatic disease. She underwent symptomatic treatment and was discharged home. In June 2016, she died due to complications of her disease.

### Discussion

According to the WHO, MPNSTs are defined as any tumor originating from a peripheral nerve or exhibiting nerve sheath differentiation. MPNST is the sixth most common type of soft tissue sarcoma [1, 2]. Approximately 50% of all MPNST cases arise sporadically, whereas the other cases are observed in patients with neurofibromatosis type 1 (NF1) [3, 4], who carry an estimated 8 to 13% lifetime risk of developing MPNST [5]. An estimated 3 to 10% of all MPNST patients have a clinical history of prior radiation exposure after a latent period of more than 15 years [3]. MPNST is typically characterized in adults with most tumors occurring in patients between 20 and 50 years of age with a median age of 35 [6].

Most MPNSTs are located along major nerve trunks, commonly arising on the body trunk, extremities, head, neck, and paravertebral regions [7]. MPNSTs arising from nerves of the small intestinal wall are extremely rare, with only fewer than 10 cases reported worldwide [8–11].

There seems to be no characteristic clinical symptoms of MPNST of the intestine. Most patients experience fatigue, weight loss, emesis, abdominal pain, and intestinal bleed [11]. Our patient presented with intestinal obstruction due to intussusception. The diagnosis is often delayed because these symptoms are usually non-specific and vague, thereby increasing the difficulty of a preoperative diagnosis of MPNST of the small intestine.

The quantitative FDG-PET imaging is used to distinguish between benign PNST and MPNST based on a tumor’s metabolic activity [12, 13]. Due to the disability to effectively confirm malignant transformation of lesions, CT and MRI are limited to define the anatomic tumor size and local invasiveness of PNST [14, 15]. The quantitative FDG-PET imaging combined with CT or MRI may be the best way to distinguish MPNST from benign PNST. However, radiographic imaging of MPNST has not supplanted histopathologic examination as the gold standard for the diagnosis of MPNST [16]. In our case, the CECT of the abdomen revealed an intussusception in the ascending colon with a smooth enhancing 3.5 cm mass suspicious for neoplasm, but could not confirm its definite lesion.

It is important to recognize that there is still a lack of widely accepted diagnostic criteria for MPNST [16]. These tumors have well-described morphological heterogeneity, and staining reveals highly cellular spindle cell tumor in fascicles [10]. S-100 protein has been the classic and most widely used antigen for documenting nerve sheath differentiation. CD34 is expressed in some MPNSTs and is likely a reflection of perineurial differentiation. Many studies suggest that elevated Ki67 expression is associated with decreased survival in MPNST [6, 17]. The reactivity of S-100 protein and high levels of p53 and Ki67 can be useful in making the final diagnosis [11]. In our case, the diffuse expression of S-100 protein and the level of Ki67 were in favor of MPNST.

Forty to 65% of MPNST patients experience local recurrence, and 30 to 60% develop metastases within 12 months of initial surgery [16]. Factors that predict recurrence include anatomic site, tumor size (≥10 cm), and adequacy of margins. Factors that predict metastases include tumor size (≥10 cm) or tumors that are American Joint Committee on Cancer stage III [16]. Over two thirds of metastases develop in the lung, whereas the other sites include the liver, brain, bone, and adrenal gland [16]. To date, there is little knowledge on MPNST of the small bowel, which is thought to have a far worse prognosis than other soft tissue sarcomas.

Because of its rarity, the optimal treatment of the small bowel MPNST is not well established [18]. Current recommendations and treatment may be based only on what is known of this tumor in other locations of the body. Complete surgical resection with wide negative margins is the current standard of care for localized MPNST and is a strong predictor of survival [16]. Adjuvant radiation therapy can been used to locally control MPNST [19]. However, small bowel MPNST may not benefit from the radiotherapy due to the location in the abdominal cavity [20]. Although chemotherapy has been carried out on gastrointestinal MPNST after surgical treatment, there are no further trials evaluating the role of chemotherapy in unresectable and metastatic tumors [11]. Recent advances in therapy have focused on targeting the molecular pathways in MPNST, but the outcomes of recently clinical trials demonstrate that further studies are needed [16, 21]. The multidisciplinary approach should be adopted to cope with these tumors.

According to the clinical and pathological characteristics, this patient was diagnosed the MPNST of the small bowel. Unfortunately, further genetic analysis was not obtained, due to patient preference as this could have been helpful for creating further treatment plans. Here, we report a rare case of MPNST of the distal ileum, which, to the best of our knowledge, was also the first Chinese case of this disease. Although MPNST arising from the small bowel is extremely rare, a surgeon should also be aware of the possibility of MPNST when dealing with intestinal obstruction or intussusception. Since patients with MPNST have very poor prognosis, it is necessary to perform a quick frozen section in the operation for a clear diagnosis. We suggest an extended radical operation in case of the fast frozen section displaying spindle cell tumors of the small bowel.

## Conclusions

To date, little is known regarding MPNST of the small bowel. We report a rare case MPNST presenting as ileum-colon intussusception. Multidisciplinary approach is essential for diagnosis and treatment of these tumors. There is no definitive guideline available for treatment of small bowel MPNST. We recommend wide excision of these tumors with very close postoperative follow-up imaging. However, the role of adjuvant radiotherapy and chemotherapy is still under debate reserved only for positive margins and recurrent tumors or when wide local excision is unfeasible.
